# Validation of a deep learning model for bone fragility detection from conventional radiographs: an international cohort study

**DOI:** 10.1016/j.eclinm.2026.103974

**Published:** 2026-05-13

**Authors:** Guillaume Gatineau, Michele De Gruttola, Karen Hind, Madeleine Davies, Diane Krueger, Neil Binkley, Martin Kužma, Juraj Payer, Giuseppe Guglielmi, Astrid Fahrleitner-Pammer, Kwang Chun, Jenny Bencardino, Alain Sherman, Kai Jones, Didier Hans

**Affiliations:** a Rheumatology Unit, Bone and Joint Department, Centre of Bone Diseases, Lausanne University Hospital & University of Lausanne, Switzerland; b Medimaps Group, Plan les Ouates, Geneva, Switzerland; c Faculty of Medicine and Health Sciences, Lancaster University, United Kingdom; d School of Medicine and Public Health, University of Wisconsin, USA; e Comenius University Faculty of Medicine, 5th Department of Internal Medicine, University Hospital, Bratislava, Slovakia; f Department of Clinical and Experimental Medicine, Foggia University School of Medicine, Italy; g Radiology Unit, Dimiccoli Hospital, Italy; h Radiology Unit, Scientific Institute Casa Sollievo della Sofferenza Hospital, Italy; i Division for Endocrinology and Diabetology, Department for Internal Medicine, Medical University Graz, Austria; j Department of Radiology, Montefiore Medical Center, The University Hospital for Albert Einstein College of Medicine, USA

**Keywords:** Artificial intelligence, Osteoporosis, Radiology, Opportunistic, Deep-learning

## Abstract

**Background:**

Artificial Intelligence (AI)-based opportunistic risk stratification solutions can help to counter rising fragility fracture rates. Existing tools estimate bone mineral density (BMD) alone, while the present study incorporates trabecular bone score (TBS), a surrogate of bone microarchitecture, more closely mirroring fracture pathophysiology. We aimed to evaluate the performance of an AI tool that estimates bone fragility directly from standard radiographs to identify individuals at highest risk of fracture.

**Methods:**

This retrospective, multinational cohort study included 18,858 paired radiographs and lumbar spine dual-energy X-ray absorptiometry (DXA) scans from adult patients (aged at least 20 years) from three clinical sites in Europe and two sites in the United States. Routine clinical radiographs of the spine, abdomen, chest, or pelvis acquired in the anteroposterior or posteroanterior view and including visualisation of the lumbar spine were included. Eligible radiographs had an in-plane spatial resolution of ≤0.2 mm per pixel, independent of vendor, and had a corresponding DXA examination within 6 months, and included at least two lumbar vertebrae (L1–L4). The AI model training used a composite Bone Fragility Index, combining TBS and BMD. Training, internal validation and testing was performed on two European sites (n = 10,692; Italy and Austria); and external validation involved three sites with ethnically diverse populations (n = 7079): Slovakia, US site 1 (Wisconsin) and US site 2 (New York). Model performance for identifying very high bone fragility (characterised by degraded TBS and osteoporosis) prioritised specificity (as per the intended clinical use to prioritise low false-positive rates) and was evaluated with accuracy, sensitivity, specificity, AUC and precision.

**Findings:**

Between Jan 1, 2010 and Dec 31, 2023, 18,858 paired radiographs and lumbar spine dual-energy X-ray absorptiometry (DXA) scans from 11,138 participants across five international sites were retrospectively aggregated. Internal testing on two European sites demonstrated an accuracy of 0.86 (95% CI: 0.78, 0.92), specificity of 0.93 (0.85, 0.99), and sensitivity of 0.53 (0.41, 0.67). External testing on three sites demonstrated consistently high specificity of 0.88 (0.77, 0.99) in the European cohort, 0.94 (0.91, 0.97) in the American White dataset, and 0.96 (0.81, 0.99) in the American Non-White. External sensitivity ranged from 0.53 (0.41, 0.67) to 0.64 (0.50, 0.88).

**Interpretation:**

The proposed approach provides rapid identification of individuals with very high bone fragility from routine radiographs. Its specificity across diverse populations supports clinical use for opportunistic osteoporosis screening in real-world settings. Future work should assess the model’s performance in more sex-balanced cohorts without prior DXA assessment, and evaluate its ability to predict incident fractures.

**Funding:**

The Swiss National Science Foundation, the Foundation of the Orthopaedic Hospital of the Vaudois University Hospital (Lausanne, Switzerland), and Medimaps Group SA, Switzerland.

**Research in context**

**Evidence before this study**

Despite the high burden of fragility fractures, a large proportion of the population at elevated fracture risk remain unscreened for osteoporosis. Opportunistic approaches using routinely acquired radiographs have emerged as a promising way to expand screening coverage. We searched PubMed and Web of Science databases (December 2020 to February 2025), building on a previous qualitative review (January 2015 to December 2020), using keywords related to osteoporosis, fragility fractures, bone mineral density, and artificial intelligence (machine and deep learning), limited to English-language publications. The search yielded 97 relevant studies developing or validating models for bone assessment, osteoporosis classification, fracture risk prediction, bone segmentation, and artificial intelligence (AI)-assisted clinical decision-making. Study quality varied, with few externally validated models and concerns regarding bias in performance evaluation. Recent advances in AI in osteoporosis have increasingly adopted multi-stage and multi-factorial approaches to enhance precision and clinical applicability, moving beyond general triage. However, most relied on bone quantity as the reference standard, typically the bone mineral density (BMD), without integrating bone quality.

**Added value of this study**

This study evaluates a multi-stage AI-driven solution that segments lumbar vertebrae and estimates bone fragility directly from standard radiographs by integrating BMD related information with trabecular bone score (TBS) into a composite Bone Fragility Index (BFI). Using a large, multinational dataset with internal and external validation across European and ethnically diverse U.S. cohorts, the model was optimised for high specificity to opportunistically identify individuals with very high BFI, defined by osteoporosis and degraded microarchitecture. The deliberate prioritisation of specificity (reflecting lower false-positive rates) over sensitivity was motivated by the intended clinical use case of opportunistic pre-dual-energy X-ray absorptiometry (DXA) screening, rather than population-wide diagnostic testing. Our approach extends prior AI methods beyond BMD-only estimation to more closely align with fracture pathophysiology.

**Implications of all the available evidence**

Taken together with existing evidence, these findings suggest that AI-based analysis of routine radiographs may enable opportunistic identification of individuals at highest bone fragility and fracture risk who might otherwise remain unscreened. By prioritising high specificity, such approaches may be well suited for integration into routine clinical workflows, where minimising unnecessary referrals is important. Incorporating microarchitectural information alongside BMD may improve risk stratification and support targeted referral for confirmatory assessment and preventive management. More research is required to assess whether this strategy could contribute to scalable osteoporosis case-finding in real-world clinical settings.

## Introduction

Osteoporosis is a major public health challenge, affecting over 500 million people worldwide and more than 10 million in the United States.^1^^,^^2^ The condition is characterised by low bone mass and bone microarchitectural deterioration, and substantially increases the risk of fragility fractures, particularly at the hip and spine, which are associated with high morbidity, mortality, and healthcare costs.^1^, ^2^, ^3^, ^4^ Nearly one-third of hip fracture patients die within a year, and the global economic burden of osteoporotic fractures exceeds $400 billion annually.^3^^,^^4^

Despite the availability of effective treatments, underdiagnosis and undertreatment remain widespread. As many as 70% of individuals at elevated fracture risk are not identified or treated, even after a primary fracture.^1^^,^^4^ This gap is particularly concerning in the context of an aging population and rising fracture incidence.^2^^,^^4^

Currently, dual-energy X-ray absorptiometry (DXA) is the standard imaging method used to assess bone mineral density (BMD), a key determinant of fracture risk. However, BMD alone does not fully reflect bone strength, as it fails to capture factors such as microarchitectural integrity, bone morphology or localised weakening. Importantly, a substantial proportion of low-trauma fractures occur in individuals with osteopenia or even normal BMD.^5^ Trabecular bone score (TBS), derived from DXA images, is an established index of bone microarchitecture that complements BMD by providing an indirect measure of bone quality, which is less affected by degenerative changes.^6^^,^^7^

Emerging artificial intelligence (AI) approaches have demonstrated potential for estimating BMD from conventional radiographs using deep learning models.^8^, ^9^, ^10^, ^11^, ^12^, ^13^, ^14^ However, most existing tools focus on BMD estimation alone without accounting for other fragility determinants such as bone microarchitecture, thereby limiting their clinical utility for comprehensive fracture risk stratification.

This study evaluates an AI-based tool designed to address this gap by opportunistically assessing bone fragility from standard radiographs with two or more lumbar vertebrae. Uniquely, it combines radiographic features with an AI-derived Bone Fragility Index based on ground-truth BMD and TBS categories from DXA, enabling the assessment of both bone mass and quality. This integrated approach supports early identification of individuals at elevated fracture risk and may prompt further evaluation with DXA or fracture risk tools such as FRAX.^15^, ^16^, ^17^, ^18^

The purpose of this study was to evaluate the internal and external performance of the model for detecting individuals with very high bone fragility, using DXA as the reference standard and data from multiple, demographically diverse international cohorts.

## Methods

### Study design

This retrospective, multinational cohort study sought to evaluate the performance of an AI-powered tool for assessing bone fragility from standard radiographs. The model was designed for integration into radiology workflows using Digital Imaging and Communications in Medicine (DICOM) compliant data. All analyses were conducted on previously acquired imaging data, with no prospective enrolment or predefined study period. Reporting and design followed the STARD checklist for diagnostic accuracy studies. A high-level study protocol shared with collaborators at study initiation is provided in the Supplementary Materials, section 3.

### Ethics

This retrospective study was conducted in accordance with the Declaration of Helsinki. Data were obtained from three centres in Europe and two centres in the USA under formal data sharing and collaboration agreements. Institutional ethical approvals and reference numbers included the University of Wisconsin Madison, Minimal Risk Research IRB (ID number 2023-0789), the Ethics Committee of the Casa Sollievo della Soffrenza Hospital in San Giovanni Rotondo (ID number 126/2023), the Montefiore Einstein Office of Human Research Affairs (amendment to IRB 2021-12959, reference 114555), and the Ethics Committee of University Hospital Bratislava (ID number EK/51/2023). For the private Radiology Graz site (Austria), formal approval from a university ethics committee was not required according to local regulations governing retrospective use of anonymised data; all data were handled in compliance with applicable ethical standards and the General Data Protection Regulation (GDPR).

At each site, DXA and radiographic examinations were de-identified prior to transfer using manufacturer software and local data protection procedures. All datasets were pseudonymised at source, keeping image data, acquisition parameters, sex, ethnicity, and birth and acquisition year. Additional safeguards were applied before centralised analysis to ensure compliance with applicable data protection regulations. Written informed consent was waived, as per the ethics review, owing to the retrospective design of the study.

### Patient selection and data sources

The study included paired radiographs and DXA examinations from adult patients above 20 years old, retrospectively aggregated from five international clinical sites in Europe and the United States. Patients were identified based on the availability of both imaging modalities within the same institution, in accordance with local ethical approvals, data protection regulations, and privacy safeguards. Data were obtained through HIPAA-compliant export procedures from imaging manufacturer software, ensuring removal of direct identifiers. Given the retrospective design, no predefined study period or target sample size was specified; inclusion was limited by the availability of eligible paired radiograph-DXA examinations at participating sites.

The training, internal validation and internal test datasets were derived from the combined Italian and Austrian clinical sites. Three additional clinical sites, including Slovakia (Comenius University Hospital), US site 1 (University of Wisconsin) and US site 2 (Montefiore, New-York) were obtained subsequently and used exclusively for external validation, reflecting heterogeneity in acquisition settings and patient populations. External validation datasets were further split by ethnicities into EU White (Slovakia), US White and US non-White to assess model generalisability.

Eligible radiographs had an in-plane spatial resolution of ≤0.2 mm per pixel, independent of vendor. From this set of standard radiographs, images were further retained if they had a corresponding DXA examination acquired within 6 months and included at least two lumbar vertebrae (L1–L4). Standard radiographs were defined as routine clinical radiographs of the spine, abdomen, chest, or pelvis acquired in the anteroposterior or posteroanterior view and including visualisation of the lumbar spine. Radiographs were further excluded if text overlays, surgical materials overlap, severe degenerations or image artifacts prevented the visualisation of at least two lumbar vertebrae. The 6-month interval between radiograph and DXA acquisition was selected to ensure both modalities were obtained within the same year, thereby minimising the potential impact of bone turnover between examinations. Vertebral compression fractures were not excluded from radiographs as vertebral fracture assessment (VFA) was not available for this study.

### Reference standard and image annotation

**Fig. 1:**
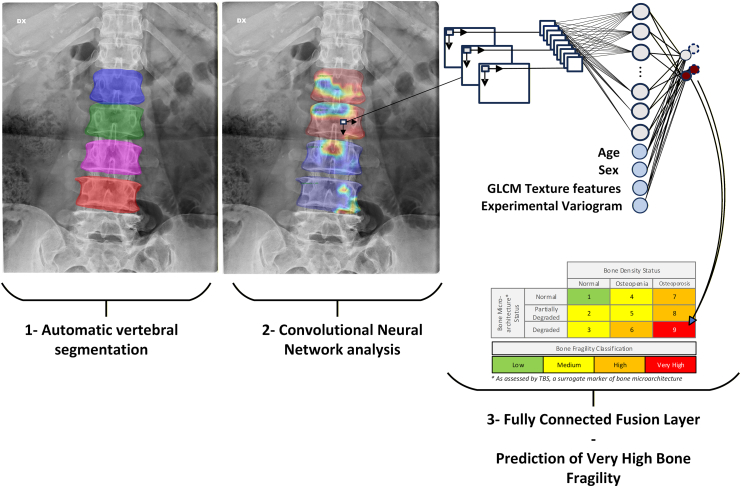
**Deep-learning multi-stage workflow.** 1. Automatic vertebral segmentation: L1–L4 vertebrae are segmented from anteroposterior or posteroanterior radiographs. Each segmented vertebra forms a region of interest, used in subsequent analysis if at least two vertebrae are present. 2. Convolutional Neural Network analysis: Lumbar spine vertebrae regions of interest are analysed by the Poolformer-s12 backbone for very high bone fragility classification. 3. Fusion-based prediction: The feature maps generated by the Poolformer-s12 are connected to two parallel fully-connected layers of size 16 each. The fully connected layers contain neural network-derived image features, and are combined with age, sex, texture metrics from the grey-level co-occurrence matrix (GLCM), and an experimental variogram serving as raw TBS to generate the very high bone fragility classification.

The reference standard or ground truth was based on a composite Bone Fragility Index (BFI) integrating lumbar spine BMD and TBS from DXA. DXA served as the reference standard modality for model training and evaluation, consistent with current clinical practice and its established role as the gold standard for osteoporosis diagnosis. The outcome of interest for model training was the “very high bone fragility” category, defined as the coexistence of osteoporosis (BMD T-score ≤ −2.5) and degraded TBS (TBS < 1.23), capturing the most severe cases of skeletal weakness (Fig. 1). These thresholds were pre-specified based on World Health Organization criteria for osteoporosis and published studies linking TBS cutoffs to elevated fracture risk.^16^, ^17^, ^18^, ^19^, ^20^ The TBS was computed using TBS Osteo Advanced (version 4.0.2), and BMD T-scores were calculated applying manufacturer normative reference curves (peak and standard deviation), using enCore v18 NHANES for GE and 2012 NHANES/BMDCS for Hologic DXA systems. When vertebrae were excluded from DXA analysis, the remaining vertebrae were referenced using the appropriate normative combination. The reference standard was then derived for all participants with available lumbar spine DXA scans, and paired with corresponding radiographs. Participants with missing or unavailable DXA or radiograph data were excluded from the analysis since both modalities are required for model training and evaluation. Information on sex/gender was obtained from DXA scan metadata as entered by the referring physician at the time of densitometry evaluation; gender identity was not available in this dataset.

All radiographs underwent standardised preprocessing, including manual segmentation of L1–L4 vertebrae using the Darwin V7 platform (V7Labs). A structured and pre-defined annotation workflow was implemented within V7, involving three independent annotators and a medical doctor as reviewer. To mitigate inter and intrareader variability, annotators followed a detailed, standardised protocol, and each segmentation was subject to independent verification. If the reviewing physician identified any discrepancies or errors—such as incorrect vertebral labelling or boundary inaccuracies—the scan was rejected with documented reasons and returned to the annotation queue for correction. Additional annotations captured the presence of text, foreign bodies, and spinal interventions.

### Deep learning workflow and validation

**Fig. 2:**
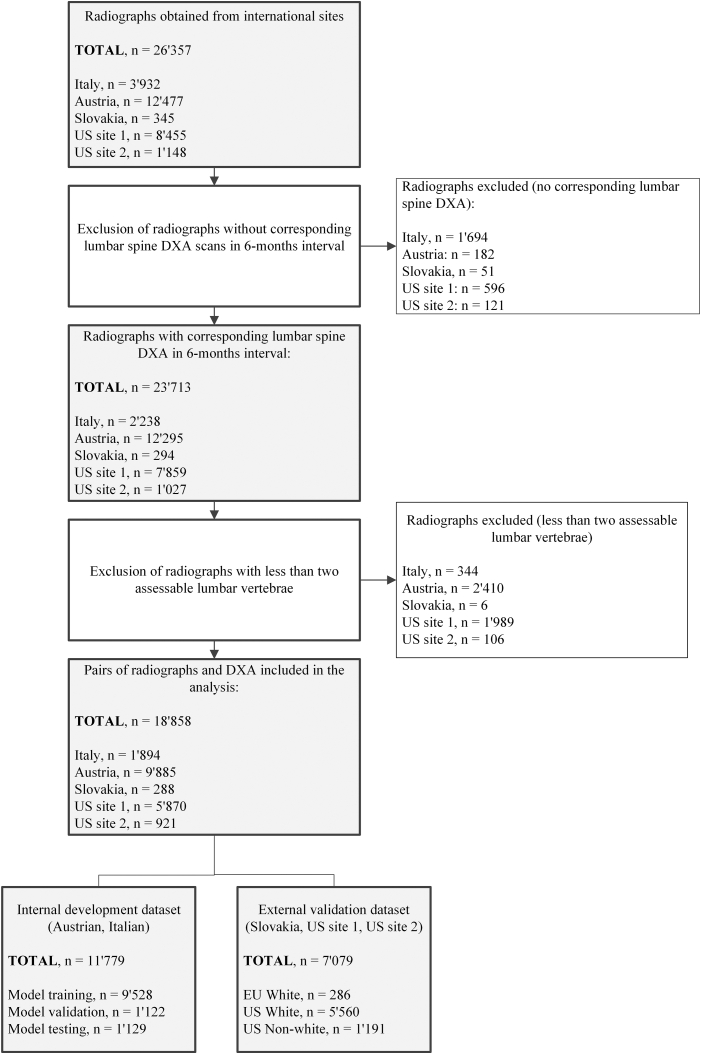
**Flow chart of participants inclusion and exclusion for all clinical sites.** US site 1: University of Wisconsin; US site 2: Montefiore, New-York; DXA: Dual-energy X-ray absorptiometry.

AI development was conducted in compliance with EU MDR and GDPR, using pseudonymised datasets and secure compute environments. Reproducibility was maintained through fixed random seeds and containerised execution environments. Training, internal validation and internal testing were conducted using a 80%-10%–10% random split of the combined Italian and Austrian datasets, stratified for the very high bone fragility class with no subject overlap to avoid data leakage (Fig. 2). The training and internal validation sets were exclusively used for model selection, model fitting, feature extraction design and hyperparameter selection. The internal test and external validation datasets were fully held out to assess the final model performance. All CNN architectures involved transfer-learning with pre-trained weights from the ImageNet-1K dataset.

The deep-learning model employed a three-stage convolutional neural network (CNN) pipeline:

i. *Vertebral Segmentation*: The first-stage CNN was designed to automatically segment lumbar vertebrae and surgical implants from abdomen, chest and pelvis anteroposterior (AP) or posteroanterior (PA) radiographs. Three CNN architectures designed for instance segmentation were trained and evaluated independently for comparative evaluation. Instance segmentation architectures were chosen over semantic segmentation, as the vertebral segmentation stage required the explicit separation of potentially overlapping lumbar vertebrae and surgical implants to enable reliable downstream inclusion or exclusion of vertebrae for bone fragility assessment. Mask R-CNN served as the baseline model due to its established performance in instance segmentation tasks. PointRend was implemented as an extension of Mask R-CNN, introducing adaptive point-based rendering to refine mask boundaries and improve contour precision. In contrast, YOLACT (You Only Look At CoefficienTs) employed a distinct strategy, generating a set of prototype masks for the entire image and combining them with per-instance coefficients predicted jointly with bounding boxes and class labels. The detailed architectures and implementations of these models have been previously described.^21^, ^22^, ^23^ The training processes involved transfer-learning from pre-trained architectures. Images were normalised to 8-bit greyscale and resized to 1024 × 1024 to ensure dimensions divisible by 32. Data augmentations included horizontal flips, brightness/contrast variation, and rotations.
ii. *Bone Fragility Classification*: The second-stage CNN was based on the Poolformer-s12 architecture to classify very high bone fragility targets including degraded TBS (TBS < 01.23) and osteoporotic lumbar spine BMD T-score (BMD T-score ≤ −2.5). PoolFormer-S12 is a lightweight MetaFormer architecture that replaces self-attention with pooling-based token mixing, reducing model complexity while enabling local and mid-range spatial aggregation.^24^ This design is well suited to capturing trabecular texture patterns relevant to bone fragility and has demonstrated competitive performance with fewer parameters, reducing overfitting risk in medical imaging settings. This architecture was extended by adding a multi-head output to the convolutional backbone, consisting of two parallel fully connected layers of 16 neurons each, integrated into both the forward and backpropagation steps, and trained to predict osteoporosis and degraded TBS. This design enabled the CNN to learn complementary representations of low bone density and impaired trabecular microarchitecture, targeting the most severe cases. As pre-processing step, a region of interest consisting of a bounding box containing all previously segmented vertebrae was used as input for classification. This approach was motivated by prior studies as opposed to using segmented and masked bone ROIs, as the surrounding pixels of a bone bring significant information to a CNN, enabling it to distinguish bone tissues from soft tissues and improving its overall performance.^25^, ^26^, ^27^ To harmonise radiographic variations across vendors and acquisition protocols, image resolution was standardised to 0.2 mm/pixel using DICOM metadata, ensuring consistent spatial scaling while preserving image quality for bone fragility classification. Histogram equalisation was applied to mitigate variability related to exposure and contrast differences across imaging systems.
iii. *Feature Fusion and Prediction*: A fusion module was added to the multi-head fully-connected layer. This fusion module enabled to enrich the CNN-extracted image features with age and sex as clinical parameters. The addition of clinical parameters have shown enhance deep-learning osteoporosis classifiers in previous studies.^10^^,^^28^, ^29^, ^30^, ^31^ In this context, age and sex values were chosen as they represent the most readily available and typical DICOM values entered from a physician during a radiograph acquisition. In addition to these clinical parameters, individual-level segmented vertebrae were used to compute texture features including grey-level co-occurrence matrix (GLCM) and an experimental variogram serving as a raw TBS score. For experimental variogram computation, histogram-normalised images were resampled to a spatial resolution of 0.45 mm/pixel. This resolution, combined with a maximum lag distance of 15 pixels, was selected to capture grey-level variations over spatial distances of approximately 6–7 mm trabecular bone. The experimental variogram was computed following the method originally described by Pothuaud et al.^32^ The variogram γ(d) quantifies spatial grey-level variability as a function of distance d and is defined as:

$$\gamma \left(d\right)=\frac{1}{2N}\sum_{i=1}^{N\left(d\right)}{\left[I\left(x_{i}\right)\;-\;I\left(x_{i}\;+\;d\right)\right]}^{2}\text{,}$$

Where I(x) represents the grey-level intensity at pixel location x, d is the distance between pixel pairs, and N(d) is the number of pixel pairs separated by distance d. The TBS feature was computed as the average of the slopes of the experimental variogram profiles, estimated on a log–log scale at a distance of 15 pixels, across all pixel pairs within each segmented vertebral ROI.

The SoftMax activation function was applied to generate final classification of very high bone fragility.

Model optimisation was performed in a configuration-driven PyTorch environment using the OpenMMLab framework. Default OpenMMLab optimisation settings were adopted, including stochastic gradient descent with a momentum of 0.9 and weight decay of 1 × 10^−4^, mixed-precision training and learning-rate scheduling with batch-size aware scaling. Grid search was employed to determine optimal hyperparameters, systematically exploring combinations of base learning rates (0.01, 0.001, 0.0001, 0.00003, 0.000001, 0.0000003) and batch sizes (16, 32, 64, 128, 256, 512, 1024).

Each experiment was fully specified via configuration files, with dynamic overrides applied at runtime to ensure reproducibility. Models were trained using automatic mixed precision when available, improving computational efficiency without compromising accuracy. Training convergence was monitored using validation loss, and early stopping was applied. Each experiment was logged and checkpointed for reproducibility.

### Statistical analysis

The study population characteristics were reported for each clinical site with mean and standard deviation for continuous variables and proportions for categorical variables. Continuous variables were compared across sites using Kruskal–Wallis rank sum tests due to potential non-normal distributions across datasets, while categorical variables were compared using Pearson’s Chi-square tests to assess differences in proportions. Segmentation models were evaluated using dedicated metrics for segmentation tasks. The mean average precision was selected to capture detection performance, while the intersection-over-union (IoU) and Dice coefficients to quantify spacial agreement between predicted and reference segmentations. The bone fragility classifier CNN performance was assessed with accuracy, sensitivity, specificity, precision, and AUC. The balanced accuracy, F1-score and Matthews correlation coefficient were computed as post-hoc analyses to further characterize model performance in the presence of class imbalance. Confidence intervals (95%) were estimated using binomial approximation, adequate for proportion-based metrics derived from binary classification outcomes. Stratified analyses by clinical site and patient ethnicity were used. No formal sensitivity analyses were conducted, as model performance was evaluated under a fixed configuration without varying key assumptions or thresholds. The formulas for classification, regression and segmentation evaluation metrics are provided in the Supplementary Tables S1–S3.

The primary fragility classifier outcome was the identification of very high bone fragility cases, corresponding to osteoporosis and degraded TBS. Post-hoc analyses introduced a high bone fragility precision metric, which quantified near-miss predictions, cases predicted as very high bone fragility but whose DXA reference fell within the high fragility zone, defined as osteopenia with degraded TBS or osteoporosis. This metric enabled to assess the precision of the model for detecting cases that would require further medical attention.

Statistical analysis was performed using R version 4.3.2. Image processing, model development, training, and validation were performed in Python (version 3.11.2), using PyTorch (version 2.0.0, CUDA 11.8) as the primary deep learning framework. Segmentation models and associated pipelines were implemented using MMDetection (version 3.0.0), with supporting computer vision utilities provided by MMCV (version 2.0.0).

### Model explainability

Explainability was assessed using Gradient-weighted Class Activation Mapping (Grad-CAM), visualising model attention within segmented vertebrae. Red hues indicated high model focus on features associated with degraded bone. Output maps were reviewed across test samples to ensure alignment with clinically relevant skeletal regions. A post hoc feature importance analysis was performed using the fully connected fusion layer of the bone fragility neural network, the engineered texture descriptors, and demographic variables as inputs to an XGBoost classifier to provide insight into the relative contribution of CNN-extracted features, engineered texture features and demographics.

### Role of the funding source

Medimaps Group SA, Switzerland, provided computational resources, access to the Darwin V7 annotation platform, and participated in data collection through collaboration agreements with participating clinical sites. The Swiss National Science Foundation and the Foundation of the Orthopaedic Hospital of the Vaudois University Hospital had no involvement in study design, data collection, data analyses, data interpretation, or the writing of the report.

## Results

### Study population

Between Jan 1, 2010 and Dec 31, 2023, 26,357 radiographs from 15,567 participants were retrospectively identified from the participating clinical sites. Images were first filtered to retain only those with a corresponding DXA examination performed within a six-month interval, yielding 23,713 paired examinations. Additional quality control criteria were applied to ensure the presence of at least two assessable lumbar vertebrae—defined as visible vertebrae in the anteroposterior or posteroanterior view without overlapping text, imaging artifacts, or surgical implants. This step excluded 4855 non-assessable radiographs, resulting in 18,858 pairs from 11,138 participants included in the final analysis. The internal dataset comprising the Italian and Austrian clinical sites was further randomly split into training, validation and test sets with stratification on the very high bone fragility category, following the 80%-10%–10% split (Fig. 2).

The mean age ranged from 62.5 years to 69.6 years (Table 1), with significant differences across sites (p < 0.001). BMI also varied between sites (p < 0.001), with the US site 2 having the highest (28.5 ± 5.8 kg/m^2^) and the Austrian the lowest (25.2 ± 4.8 kg/m^2^). TBS was consistent across sites (p = .20), while lumbar spine BMD T-scores differed (p < 0.001), with higher values in the US site 1. The prevalence of very high bone fragility ranged from 8.9% (US site 1) to 23% (Italian). External validation datasets were more ethnically diverse, particularly US site 2 (54% Hispanic, 33% Black, 5% Asian), relative to the predominantly White internal dataset. The population characteristics of the internal splits is presented in the Supplementary Materials, Table S4.

**Table 1:** Characteristics of the study population with paired Radiographs and DXA scans.

	Internal validation cohorts		External validation cohorts			p-value^b^
	Austrian N = 9885^a^	Italian N = 1894^a^	Slovakian N = 288^a^	US site 1 N = 5870^a^	US site 2 N = 921^a^	
Sex (Female) *n (%)*	9020 (91%)	1598 (84%)	254 (88%)	4720 (80%)	921 (100%)	<0.001
Sex (Male) *n (%)*	865 (9%)	296 (16%)	34 (12%)	1150 (20%)	0 (0%)	<0.001
Age (years) *mean*±*SD*	66.0 ± 10.9	62.5 ± 11.2	67.8 ± 11.2	69.6 ± 10.3	67.7 ± 9.7	<0.001
BMI (kg/m2) *mean*±*SD*	25.2 ± 4.8	27.5 ± 5.6	26.8 ± 13.5	27.9 ± 6.2	28.5 ± 5.8	<0.001
TBS (v4) *mean*±*SD*	1.252 ± 0.100	1.256 ± 0.107	1.245 ± 0.079	1.253 ± 0.094	1.248 ± 0.085	0.2
BMD T-score *mean*±*SD*	−1.5 ± 1.5	−1.5 ± 1.7	−1.4 ± 1.6	−0.3 ± 1.9	−0.9 ± 1.5	<0.001
High Bone Fragility *n (%)*	4413 (45%)	856 (45%)	119 (41%)	1459 (25%)	307 (33%)	<0.001
Very High Bone Fragility *n (%)*	1807 (18.3%)	440 (23.2%)	66 (22.9%)	522 (8.9%)	103 (11.2%)	<0.001
Ethnicity						<0.001
Asian *n (%)*	15 (0.2%)	4 (0.2%)	0 (0.0%)	135 (2.3%)	50 (5.4%)	
Black *n (%)*	0 (0.0%)	0 (0.0%)	0 (0.0%)	84 (1.4%)	307 (33.3%)	
Hispanic *n (%)*	2 (<0.1%)	0 (0.0%)	2 (0.7%)	88 (1.5%)	499 (54.2%)	
White *n (%)*	9868 (99.8%)	1890 (99.8%)	286 (99.3%)	5563 (94.8%)	65 (7.1%)	

BMI = Body Mass Index (kg/m2), TBS = Trabecular Bone Score v4.0.2, BMD = Bone Mineral Density; High Bone Fragility Index = Individuals with either densitometric osteopenia with degraded TBS or densitometric osteoporosis; Very High Bone Fragility Index = Individuals with densitometric osteoporosis and degraded TBS. Ethnicities were collected from electronic health records. p-values correspond to comparisons across sites, calculated using Kruskal–Wallis tests for continuous variables and Pearson’s chi-square tests for categorical variables.

a Mean (SD); n/N (%).

b Kruskal–Wallis rank sum test; Pearson’s Chi-squared test.

### Vertebral segmentation performance

The PointRend model was selected for integration into the AI pipeline. The final model utilised a ResNet-50 backbone with a feature pyramid network and point-based refinement, trained for 50 epochs (AdamW optimiser). In internal testing, PointRend achieved the highest mean average precision (0.897 [95% CI: 0.881, 0.913]) at an IoU threshold of 0.75, outperforming other candidates (Supplementary Materials, Table S5).

External validation on the US site 2 confirmed consistent performance, with Dice coefficients ranging from 0.822 (L1) to 0.866 (L3) and IoU values ranging from 0.815 to 0.858. Segmentation across the L1–L4 region showed a Dice score of 0.977 and an IoU of 0.960. The performance for surgical implant segmentation was an IoU of 0.925 and a Dice score of 0.962 (Supplementary Materials, Table S6).

Examinations of PointRend segmentations showed the typical cases where the model makes errors or misclassifies vertebral bodies (Supplementary Materials, Fig. S1).

### Model performance for very high bone fragility classification

The best performing Poolformer-s12 was obtained at the 38th training epoch, after which the validation accuracy did not improve by 0.005 in the 40 following epochs. The final hyperparameter settings were a learning rate of 0.0001 and a batch size of 128. The Table 2 presents the internal and external performance for bone fragility classification.

**Table 2:** Model performance for detection of very high bone fragility across internal and external cohorts.

Cohort (Ethnicity)	VHBF, n/N (%)	Accuracy (95% CI)	Sensitivity (95% CI)	Specificity (95% CI)	AUC (95% CI)
Internal test (White: AU + IT)	224/1129 (19.8%)	0.85 [0.78, 0.92]	0.53 [0.41, 0.67]	0.93 [0.85, 0.99]	0.85 [0.82, 0.89]
External test (Non-White: US sites 1 and 2)	175/1191 (14.7%)	0.90 [0.84, 0.96]	0.60 [0.43, 0.86]	0.96 [0.81, 0.99]	0.86 [0.80, 0.87]
External test (White: Slovakia)	66/286 (23.1%)	0.83 [0.71, 0.93]	0.64 [0.50, 0.88]	0.88 [0.77, 0.99]	0.86 [0.82, 0.92]
External test (White: US site 1)	453/5560 (8.1%)	0.91 [0.88, 0.93]	0.58 [0.47, 0.69]	0.94 [0.91, 0.97]	0.83 [0.76, 0.85]

AU = Austrian cohort; IT = Italian cohort; SK = Slovakian cohort; VHBF = Very High Bone Fragility; AUC = Area Under the Curve; 95% CI = 95% Confidence Interval calculated from Binomial distribution approximation.

In the internal test set, the model achieved an accuracy of 0.85 (95% CI: 0.78, 0.92), sensitivity of 0.53 (95% CI: 0.41, 0.67), and specificity of 0.93 (95% CI: 0.85, 0.99), with an AUC of 0.85 (95% CI: 0.82, 0.89). External validation in a non-White US population demonstrated strong generalisability, with an accuracy of 0.90 (95% CI: 0.84, 0.96), sensitivity of 0.60 (95% CI: 0.43, 0.86), specificity of 0.96 (95% CI: 0.81, 0.99), and AUC of 0.86 (95% CI: 0.80, 0.87). In the Slovakian external validation, model accuracy was 0.83 (95% CI: 0.71, 0.93), sensitivity 0.64 (95% CI: 0.50, 0.88), specificity 0.88 (95% CI: 0.77, 0.99), and AUC 0.86 (95% CI: 0.82, 0.92). Performance remained stable in the US site 1 White subgroup, with an accuracy of 0.91 (95% CI: 0.88, 0.93), sensitivity 0.58 (95% CI: 0.47, 0.69), specificity 0.94 (95% CI: 0.91, 0.97), and AUC 0.83 (95% CI: 0.76, 0.85). The confusion matrices for both internal and external tests are provided in the Supplementary Materials, Table S7. Balanced accuracy, F1-score, and Matthews correlation coefficient (MCC) were computed from confusion matrices (Supplementary Table S8). Balanced accuracy ranged from 0.73 (95% CI: 0.69–0.77) to 0.78 (95% CI: 0.74–0.82) across internal and external cohorts. F1-scores ranged from 0.51 (95% CI: 0.48–0.55) to 0.66 (95% CI: 0.61–0.70), and MCC values ranged from 0.47 (95% CI: 0.43–0.50) to 0.61 (95% CI: 0.56–0.65).

The precision for very high to high bone fragility was 0.94 (95% CI: 0.76, 0.99) in the internal test, and ranged from 0.86 (95% CI: 0.79, 0.95) to 0.91 (95% CI: 0.69, 0.99) in external cohorts (Supplementary Materials, Table S8).

### Model explainability

**Fig. 3:**
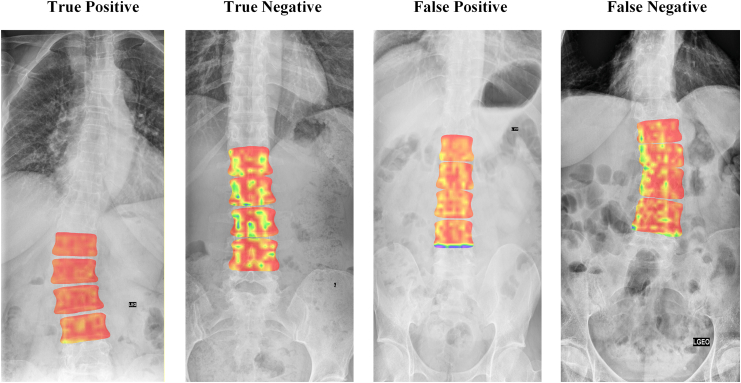
**Model explainability using gradient class activation mapping (Grad-CAM)**.

In True Positive and True Negative cases, activation maps were diffusely distributed across the trabecular bone and vertebral endplates, areas critical for bone strength assessment (Fig. 3). False Positive cases showed limited activation, often at the inferior margins of L4, while False Negative cases displayed irregular peripheral attention patterns, suggesting influence from anatomical variation or suboptimal segmentation. In contrast, True Negative examples showed low activation within vertebral segmentations.

The post hoc feature importance analysis was performed using the fully connected fusion layer, engineered texture descriptors, and demographic variables as inputs to an XGBoost classifier (Supplementary Materials, Fig. S2). This analysis showed that the raw TBS score and texture features were the most predictive of very high bone fragility. CNN-derived image features and demographic variables contributed incrementally.

## Discussion

This study presents the development and validation of a deep learning–based tool designed to assess bone fragility using existing standard radiographs. Bone fragility assessment, distinct from fracture risk assessment, was based on DXA-derived reference BMD and TBS to capture skeletal weakness. The model was trained and evaluated using 18,858 paired radiographs and DXA, and accurately predicted bone fragility indices, traditionally derived from DXA. The model demonstrated consistent external performance across all tested parameters, supporting its generalisability. Model performance metrics were aligned with the intended use of opportunistic screening in routine clinical radiography. Given the high burden of osteoporosis and limited capacity of current screening programs, specificity was prioritised to limit false positives, while preserving acceptable sensitivity and maintaining a robust AUC and precision. This focus supports the intended use of the model as an opportunistic pre-DXA triage tool (rather than population-wide diagnostic testing), to identify individuals who are truly at elevated risk and most likely to benefit from confirmatory DXA assessment. External validation cohorts yielded stable performance, with specificities between 0.85 and 0.96 and AUCs ranging from 0.83 to 0.86. Importantly, precision remained robust across validation datasets, with only a modest reduction from internal to external cohorts (0.94 to 0.86 in White European and U.S. sites; 0.91 in a non-White U.S. site). The sensitivity was moderate (0.53 to 0.64), reflecting the prioritisation of specificity in an opportunistic pre-DXA screening context. While this design ensures that most flagged individuals are truly at risk, it also means that some patients with actual bone fragility may not be identified. Importantly, these individuals remain eligible for DXA through standard clinical pathways, and missed cases may be mitigated by embedding opportunistic screening within existing frameworks such as fracture liaison services (FLS), where prior fragility fractures and other clinical risk factors independently trigger DXA referral, reducing reliance on imaging alone.

Internal cohorts were predominantly White with lower BMIs, unlike external cohorts with more diverse, urban, and multi-ethnic populations. In predictive modelling, it is generally preferable for the training population to resemble the target population to optimise performance and minimise bias. However, the current study intentionally trained on more homogeneous internal cohorts and then validated in demographically diverse external cohorts, constituting a stringent test of generalisability.

Given the burden of osteoporosis, particularly its associated healthcare costs, improving the prognosis through more effective early detection is needed. This focus on specificity ensures those flagged as elevated bone fragility may truly be at risk, enabling primary care providers to triage effectively and supporting both primary and secondary fracture prevention.

A novel feature of this approach is the combined use of BMD and TBS as ground truth. As a result, the model learns to recognise clinically severe cases, detecting osteoporosis and degraded TBS. Evidence from the Manitoba cohort and other studies indicates that individuals with both osteoporosis and degraded TBS have a substantially elevated incident fracture risk.^16^, ^17^, ^18^^,^^33^ Other emerging opportunistic osteoporosis screening tools have recently been described in the literature.^12^^,^^34^ These tools differ from the model presented in this study as they assess BMD alone. OsteoSight™ seeks to identify individuals with a low femoral neck BMD T-score (−1.0 or lower), whereas Rho provides a score from 1 to 10, corresponding to the patient’s likelihood of a low BMD T-score (−1.0 or lower), at the femoral neck or lumbar spine. An AUC of 0.834 (0.789 to 0.880), specificity of 0.852 (0.783 to 0.930), and sensitivity of 0.628 (0.538 to 0.743) has been described for OsteoSight™.^34^ Meanwhile, Rho was externally validated across three North American cohorts, finding an AUC of 0.89 (0.87 to 0.90), 0.87 (0.85 to 0.87) and 0.82 (0.79 to 0.85) for classifying low BMD.^12^ In terms of sensitivity and specificity, Rho has the highest sensitivity of 0.88 while the present study had the highest specificity, reflecting the decision to prioritise specificity and limit clinical burden during opportunistic screening.

The proposed model integrates a CNN for automated segmentation of lumbar vertebrae. CNNs are well established for their ability to capture spatial hierarchies in medical imaging.^14^ The PointRend-based architecture, selected through internal benchmarking, yielded DICE coefficients exceeding 0.82 for individual vertebrae and 0.97 for the aggregated lumbar spine region in external validation. Accurate segmentation is a prerequisite for consistent feature extraction and robust downstream classification. By eliminating the need for manual ROI annotation, automated pipelines facilitate end-to-end deployment and align with the scalability requirements of real-world radiology workflows.

Beyond architectural design, prior studies have demonstrated that incorporating clinical variables such as age and sex, can enhance predictive performance.^10^^,^^30^ In this study, we employed a hybrid modelling approach that fused deep CNN-extracted imaging features with engineered texture descriptors, including GLCM and experimental variograms, alongside patient-level metadata. This multimodal integration enabled the model to capture both local morphological patterns and broader clinical context.

This study has several strengths. It uses high-quality international datasets for both development and validation, enhancing credibility and generalisability. It applies a clinically grounded fragility definition (BMD and TBS), and includes detailed performance breakdowns across sites and ethnicities.

Limitations include that the model only analyses radiographs with two or more lumbar vertebrae, limiting applicability to spine, abdominal, chest, and pelvic imaging. Future work will extend to other sites. All participants had paired radiography and DXA examinations, reflecting a population already selected for skeletal health evaluation rather than a fully unselected opportunistic screening cohort. This was intentional for external validation of the model. Future prospective validation in radiographic cohorts without prior DXA assessment will further inform real-world performance in unselected opportunistic screening settings. The absence of fracture readings prevented the identification or annotation of vertebral fractures on radiographs, although DXA-derived reference measurements appropriately accounted for exclusions from DXA examinations. Incorporating vertebral fracture detection or confirmatory fracture assessment in future models could further broaden clinical applicability. Information on sex/gender was derived from DXA scan metadata entered during routine clinical care rather than self-report, and possible misclassification cannot be excluded. The study population had a higher prevalence of females (∼88%), reflecting the typical population undergoing osteoporosis screening and densitometry, particularly post-menopausal women who represent the highest-risk group for osteoporosis. Nevertheless, this imbalance warrants further validation in more sex-balanced cohorts. While optimised for detecting very high bone fragility, the model does not estimate absolute fracture risk or time to fracture, which are needed for full prognostic utility. Future studies will expand clinical applicability by evaluating fracture prediction capabilities and the independent contributions of age and sex on model performance.

Reproducibility across repeated scans and vendors needs further testing, though segmentation consistency suggests reliable localisation.

This study presents an AI-based approach for assessing bone fragility directly from standard radiographs. The model demonstrated high specificity and maintained performance across external cohorts, indicating potential for opportunistic screening to identify individuals at increased bone fragility. By enabling opportunistic screening from radiographs already acquired for other clinical purposes, the present solution facilitates early identification of high-risk individuals and optimised patient triage. This approach could also streamline secondary fracture prevention pathways while addressing real-world limitations in DXA accessibility. Future work will include prospective validation, evaluation within fracture liaison services, and extension to other anatomical regions to further support its clinical applicability.

## Contributors

Guillaume Gatineau contributed to the literature search, study design, figure preparation, data collection, data curation, data analysis, data interpretation, and drafting of the original manuscript. Michele De Gruttola contributed to figure editing, study design, data collection, data curation, data analysis, data interpretation, and manuscript review and editing. Karen Hind contributed to the literature search and to drafting, review, and editing of the manuscript. Madeleine Davies contributed to the literature search and to manuscript review and editing. Diane Krueger and Neil Binkley contributed to data collection, data curation, and manuscript review and editing. Martin Kužma, Juraj Payer, Giuseppe Guglielmi, Astrid Fahrleitner-Pammer, Kwang Chun, Jenny Bencardino, and Alain Sherman contributed to data collection and manuscript review and editing. Kai Jones contributed to data collection, data curation, and manuscript review and editing. Didier Hans contributed to study design and to drafting, review, and editing of the manuscript. Guillaume Gatineau and Diane Krueger accessed and verified the underlying data.

## Data sharing statement

The data used in this study are proprietary and subject to restrictions from academic–industry collaboration agreements and data protection regulations; therefore, they cannot be shared in full. However, a set of de-identified sample radiographs with associated annotations will be made available by the corresponding author upon reasonable request for independent validation and community benchmarking. A high-level study protocol shared with collaborators at study initiation is provided in the Supplementary Materials, section 3. The software and source code used in this study cannot be shared in full, as they form part of a regulated proprietary software as a medical device developed by Medimaps Group SA. As a result, public release of the complete codebase is not permitted. Relevant methodological details are provided in the manuscript to support transparency and reproducibility.

## Declaration of interests

Diane Krieger, Neil Binkley, Martin Kužma, Juraj Payer, Giuseppe Guglielmi, Astrid Fahrleinter-Pammer, Kwang Chun, Jenny Bencardino, Kai Jones and Alain Sherman declare that they have no conflicts of interest associated with publication of this study. Guillaume Gatineau, Michele De Gruttola, Karen Hind and Madeleine Davies are employees of Medimaps Group SA, developers of TBS iNsight™ and TBS Reveal™ software. Didier Hans and Guillaume Gatineau are co-authors of the TBS and TBS Reveal patents. Didier Hans has corresponding shares, and is part-time CEO at Medimaps Group. CE marking and FDA clearance for TBS Reveal™ are currently pending.

## References

[1] Wright N.C., Looker A.C., Saag K.G. (2014). The recent prevalence of osteoporosis and low bone mass in the United States based on bone mineral density at the femoral neck or lumbar spine. J Bone Miner Res.

[2] Shen Y., Huang X., Wu J. (2022). The global burden of osteoporosis, low bone mass, and its related fracture in 204 countries and territories, 1990-2019. Front Endocrinol.

[3] Guzon-Illescas O., Perez Fernandez E., Crespí Villarias N. (2019). Mortality after osteoporotic hip fracture: incidence, trends, and associated factors. J Orthop Surg.

[4] Hernlund E., Svedbom A., Ivergård M. (2013). Osteoporosis in the European Union: medical management, epidemiology and economic burden: a report prepared in collaboration with the International Osteoporosis Foundation (IOF) and the European Federation of Pharmaceutical Industry Associations (EFPIA). Arch Osteoporos.

[5] Schuit S.C.E., Van Der Klift M., Weel A.E.A.M. (2004). Fracture incidence and association with bone mineral density in elderly men and women: the Rotterdam Study. Bone.

[6] Shevroja E., Reginster J.Y., Lamy O. (2023). Update on the clinical use of trabecular bone score (TBS) in the management of osteoporosis: results of an expert group meeting organized by the European Society for Clinical and Economic Aspects of Osteoporosis, Osteoarthritis and Musculoskeletal Diseases (ESCEO), and the International Osteoporosis Foundation (IOF) under the auspices of WHO Collaborating Center for Epidemiology of Musculoskeletal Health and Aging. Osteoporos Int.

[7] Hayden A.C., Binkley N., Krueger D., Bernatz J.T., Kadri A., Anderson P.A. (2022). Effect of degeneration on bone mineral density, trabecular bone score and CT hounsfield unit measurements in a spine surgery patient population. Osteoporos Int.

[8] Zhang B., Yu K., Ning Z. (2020). Deep learning of lumbar spine X-ray for osteopenia and osteoporosis screening: a multicenter retrospective cohort study. Bone.

[9] Jang R., Choi J.H., Kim N., Chang J.S., Yoon P.W., Kim C.H. (2021). Prediction of osteoporosis from simple hip radiography using deep learning algorithm. Sci Rep.

[10] Mao L., Xia Z., Pan L. (2022). Deep learning for screening primary osteopenia and osteoporosis using spine radiographs and patient clinical covariates in a Chinese population. Front Endocrinol.

[11] Sato Y., Yamamoto N., Inagaki N. (2022). Deep learning for bone mineral density and T-Score prediction from chest X-rays: a multicenter study. Biomedicines.

[12] Bilbily A., Syme C.A., Adachi J.D. (2024). Opportunistic screening of low bone mineral density from standard X-Rays. J Am Coll Radiol.

[13] Golestan K., Syme C.A., Bilbily A. (2023). Approximating femoral neck bone mineral density from hand, knee, and pelvis X-rays using deep learning. J Med Artif Intell.

[14] Gatineau G., Shevroja E., Vendrami C. (2024). Development and reporting of artificial intelligence in osteoporosis management. J Bone Miner Res.

[15] Hans D., Barthe N., Boutroy S., Pothuaud L., Winzenrieth R., Krieg M.A. (2011). Correlations between trabecular bone score, measured using anteroposterior dual-energy X-Ray absorptiometry acquisition, and 3-Dimensional parameters of bone microarchitecture: an experimental study on human cadaver vertebrae. J Clin Densitom.

[16] Boutroy S., Hans D., Sornay-Rendu E., Vilayphiou N., Winzenrieth R., Chapurlat R. (2013). Trabecular bone score improves fracture risk prediction in non-osteoporotic women: the OFELY study. Osteoporos Int.

[17] Briot K., Paternotte S., Kolta S. (2013). Added value of trabecular bone score to bone mineral density for prediction of osteoporotic fractures in postmenopausal women: the OPUS study. Bone.

[18] Popp A.W., Meer S., Krieg M.A., Perrelet R., Hans D., Lippuner K. (2016). Bone mineral density (BMD) and vertebral trabecular bone score (TBS) for the identification of elderly women at high risk for fracture: the SEMOF cohort study. Eur Spine J.

[19] Nuti R., Brandi M.L., Checchia G. (2019). Guidelines for the management of osteoporosis and fragility fractures. Intern Emerg Med.

[20] McCloskey E.V., Odén A., Harvey N.C. (2016). A meta-analysis of trabecular bone score in fracture risk prediction and its relationship to FRAX. J Bone Miner Res.

[21] He K., Gkioxari G., Dollar P., Girshick R. (2020). Mask R-CNN. IEEE Trans Pattern Anal Mach Intell.

[22] Kirillov A., Wu Y., He K., Girshick R. (2020). 2020 IEEE/CVF conference on computer vision and pattern recognition (CVPR).

[23] Bolya D., Zhou C., Xiao F., Lee Y.J. (2019). 2019 IEEE/CVF international conference on computer vision (ICCV).

[24] Yu W., Luo M., Zhou P. (2022). 2022 IEEE/CVF conference on computer vision and pattern recognition (CVPR).

[25] Ho C.S., Chen Y.P., Fan T.Y. (2021). Application of deep learning neural network in predicting bone mineral density from plain X-ray radiography. Arch Osteoporos.

[26] Kong S.H., Lee J.W., Bae B.U. (2022). Development of a spine X-Ray-Based fracture prediction model using a deep learning algorithm. Endocrinol Metab Seoul Korea.

[27] Kang J.W., Park C., Lee D.E., Yoo J.H., Kim M.W. (2022). Prediction of bone mineral density in CT using deep learning with explainability. Front Physiol.

[28] Chen Z., Luo W., Zhang Q. (2021). 2021 43rd Annual International Conference of the IEEE Engineering in Medicine & Biology Society (EMBC).

[29] Luo W., Chen Z., Zhang Q. (2022). Osteoporosis diagnostic model using a multichannel convolutional neural network based on quantitative ultrasound radiofrequency signal. Ultrasound Med Biol.

[30] Sukegawa S., Fujimura A., Taguchi A. (2022). Identification of osteoporosis using ensemble deep learning model with panoramic radiographs and clinical covariates. Sci Rep.

[31] Yamamoto N., Sukegawa S., Yamashita K. (2021). Effect of patient clinical variables in osteoporosis classification using hip X-rays in deep learning analysis. Med Kaunas Lith.

[32] Pothuaud L., Carceller P., Hans D. (2008). Correlations between grey-level variations in 2D projection images (TBS) and 3D microarchitecture: applications in the study of human trabecular bone microarchitecture. Bone.

[33] Hans D., Goertzen A.L., Krieg M.A., Leslie W.D. (2011). Bone microarchitecture assessed by TBS predicts osteoporotic fractures independent of bone density: the manitoba study. J Bone Miner Res.

[34] Pignolo R.J., Connell J.J., Briggs W. (2025). Opportunistic assessment of osteoporosis using hip and pelvic X-rays with OsteoSight^TM^: validation of an AI-based tool in a US population. Osteoporos Int.

