# Supplementary material for: Validation of a deep learning model for bone fragility detection from conventional radiographs: an international cohort study
Source: eClinicalMedicine. 2026 May 13;95:103974. doi: 10.1016/j.eclinm.2026.103974 (PMC13196392; doi:10.1016/j.eclinm.2026.103974)
Supplement: Supplementary Materials, Figs. S1 and S2, and Tables S1–S9 [file mmc1.docx]

**Supplementary Materials**

**Multinational Validation of a Deep Learning Model for Bone Fragility Detection from X-ray**

Table of Contents

[**1-** **Supplementary Figures** 2](#_Toc226541729)

[**Supplementary Figure 1**: **Visualizations of PointRend segmentation results on radiographs, showing both successful and erroneous cases** 2](#_Toc226541730)

[**Supplementary Figure 2: Post-hoc analysis of the importance of CNN-derived image features, texture parameters, gray-level co-occurrence matrix and demographic parameters used in fusion layer in very high bone fragility classification.** 3](#_Toc226541731)

[**2-** **Supplementary Tables** 4](#_Toc226541732)

[**Supplementary Table 1: Evaluation metrics for classification tasks** 4](#_Toc226541733)

[**Supplementary Table 2: Evaluation metrics for segmentation tasks** 5](#_Toc226541734)

[**Supplementary Table 3: Evaluation metrics for regression tasks** 6](#_Toc226541735)

[**Supplementary Table 4: Internal dataset characteristics from the Italian and Austrian clinical sites, stratified by training, validation, and test splits** 7](#_Toc226541736)

[**Supplementary Table 5: Comparison of the Mask R-CNN, PointRend and Yolact AI segmentation architectures using the internal test sets** 8](#_Toc226541737)

[**Supplementary Table 6: External Validation of the PointRend Segmentation Model** 8](#_Toc226541738)

[**Supplementary Table 7: Confusion matrices of the AI model by DXA ground-truth for very high bone fragility.** 9](#_Toc226541739)

[**Supplementary Table 8: Sub-evaluation of the model for precision of near-miss cases.** 9](#_Toc226541740)

[**Supplementary Table 9: Model performance for very high bone fragility classification using performance metrics accounting for class imbalance** 10](#_Toc226541741)

[**3-** **TBS Reveal – Project scope** 11](#_Toc226541742)

# **Supplementary Figures**

## **Supplementary Figure 1**: **Visualizations of PointRend segmentation results on radiographs, showing both successful and erroneous cases**


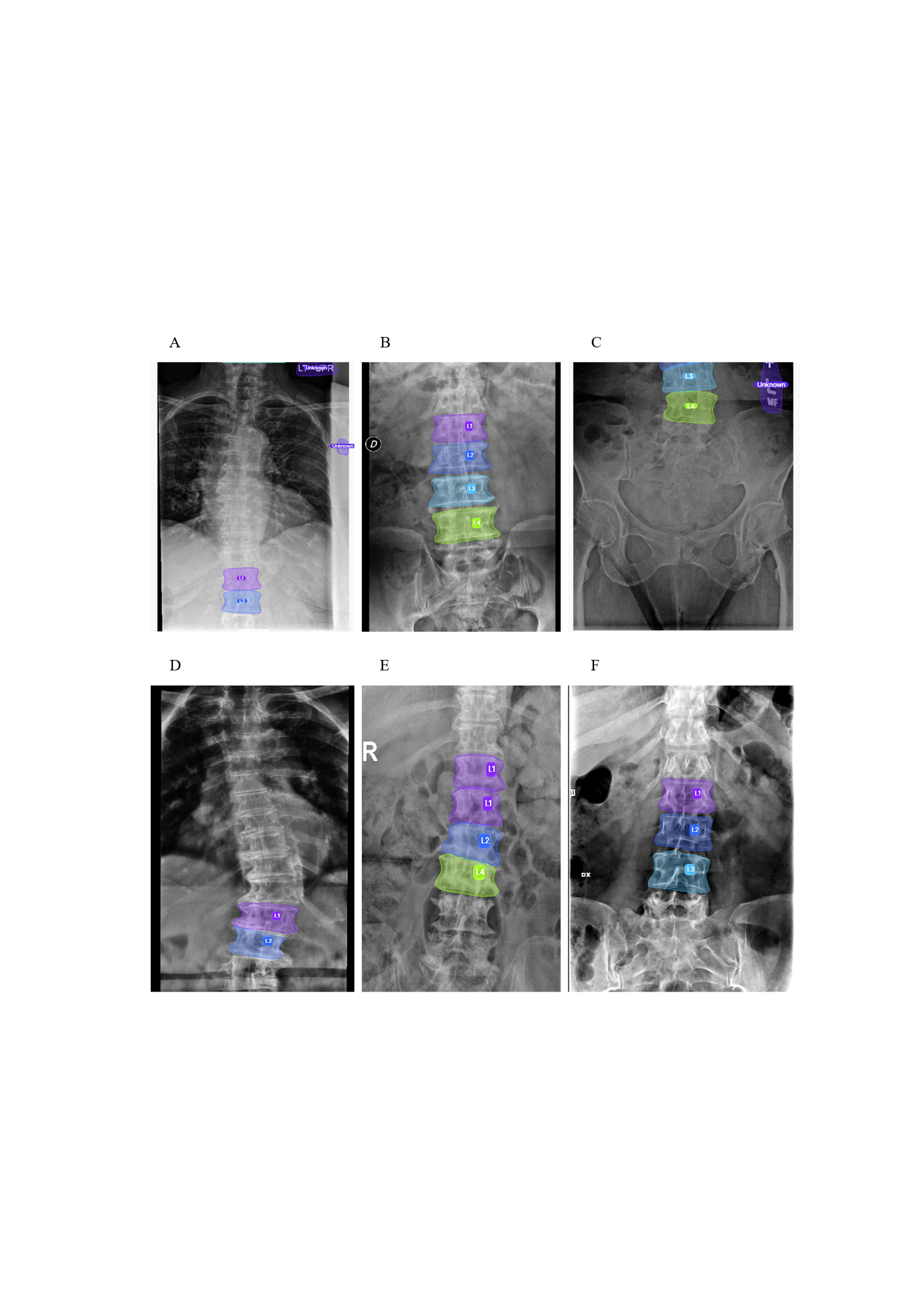

A. Accurate segmentation of L1 and L2 on a chest radiograph.
B. Accurate segmentation of L1 to L4 on an anteroposterior (AP) spine radiograph.
C. Accurate segmentation of L3 and L4 on a pelvic radiograph.
D. Erroneous segmentation on a chest radiograph, where the L3 vertebra was missed by the model.
E. Erroneous segmentation and classification, with two regions incorrectly labeled as L1.
F. Incomplete segmentation, where only three lumbar vertebrae were detected, missing L4.

## **Supplementary Figure 2: Post-hoc analysis of the importance of CNN-derived image features, texture parameters, gray-level co-occurrence matrix and demographic parameters used in fusion layer in very high bone fragility classification.**

Features of the CNN fusion layer for very high bone fragility classification include meanxtbs: experimental variogram averaged on segmented vertebrae, meanxtbsnonoisevalues: experimental variogram averaged on segmented vertebrae using a gaussian denoised image, meanxnoiseinsidevalues: average gaussian noise on segmented vertebrae, meanxnoiseoutsidevalues: average gaussian noise outside segmented lumbar spine vertebrae (surrounding lumbar area) meanxpvmeanvalues: average pixel value from segmented vertebrae, meanxpvstdvalues: average standard deviation of the pixel value from segmented vertebrae, meanglcm: gray-level co-occurrence matrix parameters averaged on segmented vertebrae, cnn2_TBS_feat and cnn2_BMD_feat: 32 CNN-extracted image features, Sex_FO_M1 binarized sex

# **Supplementary Tables**

## **Supplementary Table 1: Evaluation metrics for classification tasks**

| **TASK** | **Metric** | **Formula** | **Purpose** |
| --- | --- | --- | --- |
| **Classification tasks** | **Accuracy - PPV** | $\frac{TP+TN}{TP+TN+FP+FN}$ | Percentage of correctly predicted labels among all the predictions |
|  | **NPV** | $\frac{TN}{TN+FN}$ | Proportion of true negative predictions out of the total negative predictions. Indicator of how well a model can correctly identify negative cases. |
|  | **Precision** | $\frac{TP}{TP+FP}$ | Proportion of correctly predicted positive instances out of the total instances predicted as positive. Precision is useful in scenarios where false positives have significant consequences. |
|  | **Recall - Sensitivity** | $\frac{TP}{TP+FN}$ | Proportion of correctly predicted positive instances out of all actual positive instances. It emphasizes the ability of a model to identify positive instances correctly. Recall (or sensitivity) is essential in situations where false negatives have significant consequences. |
|  | **Specificity** | $\frac{TN}{FP+TN}$ | Proportion of correctly predicted negative instances out of all actual negative instances. It highlights the ability of a model to identify negative instances correctly. Specificity is valuable when it is crucial to avoid false positives. |
|  | **Balanced Accuracy** | $\frac{Sensitivity+Specificity}{2}$ | Average of sensitivity and specificity, providing an unbiased estimate of classification performance under class imbalance by giving equal weight to positive and negative classes. |
|  | **F1-Score** | $2*\frac{Precision*Recall}{Precision+Recall}$ | The F1-Score combines precision and recall into a single metric. It provides a balanced measure by calculating the harmonic mean of precision and recall. F1-Score is useful when both false positives and false negatives need to be minimized simultaneously. |
|  | **Matthews Correlation Coefficient (MCC)** | $\frac{TP*TN-FP*FN}{\sqrt{\left( TP+FP \right)*\left( TP+FN \right)*\left( TN+FP \right)*(TN+FN)}}$ | Summary measure of prediction agreement that incorporates true and false positives and negatives, suitable for imbalanced datasets. |
|  | **AUC-ROC** | Area under the curve of the TP rate against FP rate | Commonly used for binary classification problems, AUC-ROC quantifies the model's ability to distinguish between positive and negative instances by plotting the true positive rate against the false positive rate. Commonly used metric to represent the overall model performance. |
|  | **AUC-PR** | Area under the curve of the Precision against Recall | The Area Under the Precision-Recall Curve (AUC-PR) is another metric used for binary classification tasks. It evaluates the trade-off between precision and recall at various probability thresholds. AUC-PR is particularly useful when dealing with imbalanced datasets where the positive class is rare or when the focus is on the performance of the positive class. |

## **Supplementary Table 2: Evaluation metrics for segmentation tasks**

| **TASK** | **Metric** | **Formula** | **Purpose** |
| --- | --- | --- | --- |
| **Segmentation tasks** | **Accuracy - PPV** | $\frac{TP+TN}{TP+TN+FP+FN}$ | Percentage of correctly predicted labels among all the predictions |
|  | **NPV** | $\frac{TN}{TN+FN}$ | Proportion of true negative predictions out of the total negative predictions. Indicator of how well a model can correctly identify negative cases. |
|  | **Precision** | $\frac{TP}{TP+FP}$ | Proportion of correctly predicted positive instances out of the total instances predicted as positive. Precision is useful in scenarios where false positives have significant consequences. |
|  | **Mean Average Precision (mAP)** | $\frac{1}{n}\sum_{k=1}^{k=n} AP_{k}$ | Average of the Average Precisions (APs) calculated for each individual class. |
|  | **Recall – Sensitivity** | $\frac{TP}{TP+FN}$ | Proportion of correctly predicted positive instances out of all actual positive instances. It emphasizes the ability of a model to identify positive instances correctly. Recall (or sensitivity) is essential in situations where false negatives have significant consequences. |
|  | **Specificity** | $\frac{TN}{FP+TN}$ | Proportion of correctly predicted negative instances out of all actual negative instances. It highlights the ability of a model to identify negative instances correctly. Specificity is valuable when it is crucial to avoid false positives. |
|  | **F1-Score** | $2*\frac{Precision*Recall}{Precision+Recall}$ | The F1-Score combines precision and recall into a single metric. It provides a balanced measure by calculating the harmonic mean of precision and recall. F1-Score is useful when both false positives and false negatives need to be minimized simultaneously. |
|  | **AUC-ROC** | Area under the curve of the TP rate against FP rate | The Area Under the Receiver Operating Characteristic Curve (AUC-ROC) is commonly used for binary classification problems. It quantifies the model's ability to distinguish between positive and negative instances by plotting the true positive rate against the false positive rate. Commonly used metric to represent overall performance. |
|  | **AUC-PR** | Area under the curve of the Precision against Recall | The Area Under the Precision-Recall Curve (AUC-PR) is another metric used for binary classification tasks. It evaluates the trade-off between precision and recall at various probability thresholds. AUC-PR is particularly useful when dealing with imbalanced datasets where the positive class is rare or when the focus is on the performance of the positive class. |
|  | **Intersection over Union (IoU) – Jaccard Index** | $\frac{TP}{TP+FP+FN}$ | IoU measures the overlap between the predicted and true regions of interest in tasks such as object detection or image segmentation. It is calculated by dividing the intersection area by the union area of the two regions. IoU provides a measure of the spatial accuracy of the model's predictions. |
|  | **Mean Intersection over Union (mIoU)** | $\frac{1}{n_{c}}\sum_{i=1}^{n_{c}} {IoU}_{c_{i}}$ | mIoU is the average of IoU scores calculated for multiple classes or regions. It is widely used in tasks involving multiple object or instance segmentation. mIoU provides an overall measure of segmentation accuracy across different classes. |
|  | **Dice Index** | $\frac{2*TP}{2*TP+FP+FN}$ | The Dice Index is similar to IoU and measures the overlap between the predicted and true regions. Dice Index is advantageous when the dataset is highly imbalanced and less sensitive to class prevalence than IoU. |

## **Supplementary Table 3: Evaluation metrics for regression tasks**

| **TASK** | **Metric** | **Formula** | **Purpose** |  |
| --- | --- | --- | --- | --- |
| **Regression tasks** | **Mean Squared Error (MSE)** | $\frac{1}{n}\sum_{i=1}^{n} {{(y}_{i}-ŷ_{i})}^{2}$ | MSE calculates the average squared difference between the predicted and true values. It is commonly used in regression tasks and provides a measure of the average magnitude of the model's errors. MSE penalizes larger errors more than smaller ones. |  |
|  | **Mean Absolute Error (MAE)** | $\frac{1}{n}\sum_{i=1}^{n} \vert y_{i}-ŷ_{i}\vert$ | MAE is another widely used metric for regression models. It calculates the average of the absolute differences between the predicted and true values. Unlike MSE, MAE does not square the differences, making it less sensitive to outliers. MAE provides a measure of the average absolute magnitude of the errors. It is useful when the focus is on the average magnitude of errors and outliers should not have a disproportionate influence on the evaluation. |  |
|  | **Coefficient of determination (CD), R2** | $1-\frac{\sum_{i=1}^{n} {{(y}_{i}-ŷ_{i})}^{2}}{\sum_{i=1}^{n} {{(y}_{i}-ȳ)}^{2}}$ | R2, or the coefficient of determination, is a metric commonly used in regression analysis. It measures the proportion of the variance in the dependent variable (target) that can be explained by the independent variables (features) used in the model. R^2 ranges from 0 to 1, with a higher value indicating a better fit of the model to the data. R^2 provides an indication of how well the model captures the underlying patterns and variability in the data. It is valuable for assessing the overall goodness of fit of a regression model. |  |
|  |  |  |  |  |
|  | **Calibration** | Plot of the predicted outcome against the ground truth | Calibration refers to the alignment between predicted probabilities or confidence scores and the true probability of an event occurring. In other terms, it allows to assess how closely the points lie against identity (45-degree line) |  |

## **Supplementary Table 4: Internal dataset characteristics from the Italian and Austrian clinical sites, stratified by training, validation, and test splits**

| **Variable** | Training set, N = 9'528 *^1^* | Validation (tuning) set, N = 1’122 *^1^* | Test set, N = 1'129 *^1^* | p-value *^2^* |
| --- | --- | --- | --- | --- |
| **Age (years)** mean (SD) | 65·436 (10·987) | 65·803 (11·190) | 65·676 (11·473) | 0·3 |
| **Sex (F)** n (%) | 8’581 / 9’528 (90%) | 1’021 / 1’122 (91%) | 1’016 / 1’129 (90%) | 0·7 |
| **BMI (kg/m²)** mean (SD) | 25·602 (4·934) | 25·603 (4·981) | 25·721 (5·302) | >0·9 |
| **TBS (GT)** mean (SD) | 1·253 (0·101) | 1·247 (0·104) | 1·250 (0·097) | 0·076 |
| **TBS category** |  |  |  | 0·087 |
| **Degraded** n (%) | 3’950 / 9’528 (41%) | 505 / 1’122 (45%) | 486 / 1’129 (43%) |  |
| **Normal** n (%) | 2’668 / 9’528 (28%) | 303 / 1’122 (27%) | 282 / 1’129 (25%) |  |
| **Partially-Degraded** n (%) | 2’910 / 9’528 (31%) | 314 / 1’122 (28%) | 361 / 1’129 (32%) |  |
| **Lumbar Spine BMD T-score** mean (SD) | −1.500 (1·546) | −1·472 (1·587) | −1·556 (1·420) | 0·7 |
| **BMD T-score category** |  |  |  | 0·5 |
| **Normal** n (%) | 2’850 / 9’528 (30%) | 348 / 1’122 (31%) | 316 / 1’129 (28%) |  |
| **Osteopenia** n (%) | 4’002 / 9’528 (42%) | 483 / 1’122 (43%) | 474 / 1’129 (42%) |  |
| **Osteoporosis** n (%) | 2’676 / 9’528 (28%) | 291 / 1’122 (26%) | 339 / 1’129 (30%) |  |
| **High Fragility** n (%) | 4’222 / 9’528 (44%) | 516 / 1’122 (46%) | 531 / 1’129 (47%) | 0·15 |
| **Very High Fragility** n (%) | 1’808 / 9’528 (19%) | 213 / 1’122 (19%) | 226 / 1’129 (20%) | 0·7 |
| *^1^ Mean (SD); n / N (%)* | | | | |
| *^2^ Kruskal-Wallis rank sum test; Pearson’s Chi-squared test* | | | | |

## **Supplementary Table 5: Comparison of the Mask R-CNN, PointRend and Yolact AI segmentation architectures using the internal test sets**

|  | **AI Segmentation Architectures – Internal Test** | | |
| --- | --- | --- | --- |
|  | **Mask R-CNN** | **PointRend** | **Yolact** |
| Processing time per X-ray in seconds | 0·083s | 0·090s | 0·066s |
| Mean average precision (mAP) using 75% IoU threshold | 0·868 [0·852, 0·884] | 0·897 [0·881, 0·913] | 0·843 [0·827, 0,859] |

## **Supplementary Table 6: External Validation of the PointRend Segmentation Model**

| **Region** | **Average**  **IoU** | **Average**  **DICE** | **ap@0·5** | **ap@0·75** | [**ap@0·9**](mailto:ap@0.9) |
| --- | --- | --- | --- | --- | --- |
| **L1** | 0·8149 | 0·8223 | 0·8274 | 0·8274 | 0·8260 |
| **L2** | 0·8450 | 0·8531 | 0·8560 | 0·8560 | 0·8560 |
| **L3** | 0·8577 | 0·8659 | 0·8681 | 0·8681 | 0·8654 |
| **L4** | 0·8444 | 0·8516 | 0·8569 | 0·8556 | 0·8515 |
| **LS** | 0·9600 | 0·9774 | 1·0000 | 0·9352 | 0·8731 |
| **Surgical Implant** | 0·925 | 0·962 | 0·992 | 0·971 | 0·906 |

L1 to L4: Lumbar vertebrae; LS: Lumbar Spine; IoU: Intersection over Union; ap: Average Precision; @x: Threshold

## **Supplementary Table 7: Confusion matrices of the AI model by DXA ground-truth for very high bone fragility.**

| **Cohort** | **Ref (DXA) / Pred (AI)** | **VHBF** | **non-VHBF** |
| --- | --- | --- | --- |
| **Internal Test (White: AU + IT)** | VHBF | 119 | 105 |
|  | non-VHBF | 63 | 842 |
|  | | | |
| **External Test (Non-White: US 1–2)** | VHBF | 105 | 70 |
|  | non-VHBF | 40 | 976 |
|  | | | |
| **External Test (White: Slovakia)** | VHBF | 42 | 24 |
|  | non-VHBF | 25 | 195 |
|  | | | |
| **External Test (White: US 1)** | VHBF | 263 | 190 |
|  | non-VHBF | 310 | 4,797 |

VHBF: Very High Bone Fragility, combining osteoporosis and degraded TBS
AU: Austrian cohort (internal test)
IT: Italian cohort (internal test)

## **Supplementary Table 8: Sub-evaluation of the model for precision of near-miss cases.**

| **Cohort**  **(Ethnicity)** | **VHBF, n/N (%)** | **VHBF to High Fragility Precision**  **(95% CI)** |
| --- | --- | --- |
| **Internal Test (White: AU + IT)** | 224 /1,129 (20%) | 0.94 [0.76, 0.99] |
| **External Test (Non-White: US sites 1 and 2)** | 175 /1,191 (15%) | 0.91 [0.69, 0.99] |
| **External Test (White: Slovakia)** | 66 / 286 (23%) | 0.86 [0.68, 0.99] |
| **External Test (White: US site 1)** | 453 /5,560 (8.1%) | 0.86 [0.79, 0.95] |

VHBF: Very High Bone Fragility, osteoporosis and degraded TBS.
AU: Austrian cohort (internal test)
IT: Italian cohort (internal test)

## **Supplementary Table 9: Model performance for very high bone fragility classification using performance metrics accounting for class imbalance**

| **Cohort** | **Balanced Accuracy (95% CI)** | **F1-score (95% CI)** | **MCC (95% CI)** |
| --- | --- | --- | --- |
| **Internal Test (White: AU + IT)** | 0·73 (0·69–0·77) | 0·59 (0·54–0·63) | 0·50 (0·45–0·55) |
| **External Test (Non-White: US 1–2)** | 0·78 (0·74–0·82) | 0·66 (0·61–0·70) | 0·61 (0·56–0·65) |
| **External Test (White: Slovakia)** | 0·76 (0·70–0·82) | 0·63 (0·57–0·69) | 0·52 (0·45–0·59) |
| **External Test (White: US 1)** | 0·76 (0·73–0·79) | 0·51 (0·48–0·55) | 0·47 (0·43–0·50) |

VHBF: Very High Bone Fragility, combining osteoporosis and degraded TBS
MCC: Matthews correlation coefficient
AU: Austrian cohort (internal test)
IT: Italian cohort (internal test)

# **TBS Reveal – Project scope**


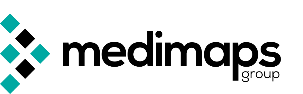
**
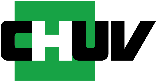
**

**MULTI-CENTRE COLLABORATIVE PROJECT SCOPE**

**Opportunistic Screening of Bone Fragility from conventional radiographs**

**Background**

Up to 37 million fragility fractures happen in the world annually, a number expected to increase given the ageing population [1]. After the age of 50 years, one in two women and one in four men will suffer a major osteoporotic fracture in their remaining lifetime [3], which results in significant direct and indirect individual costs and economic burden [4]. The great majority of individuals at high risk, including those who have already had a prior osteoporotic fracture are neither identified nor treated [6] [7] [8]. Therefore, additional approaches to identify at risk individuals are needed.

Osteoporosis is a disease characterized by low bone mineral density (BMD) and microarchitectural deterioration of bone tissue, leading to enhanced bone fragility and a consequent increase in fracture risk [5]. DXA is the current gold standard technique used for osteoporosis diagnosis and follow-up. BMD and a proxy of bone microarchitecture, trabecular bone score (TBS) can be currently assessed from the DXA images. TBS predicts osteoporotic fractures independently of BMD and clinical risk factors. The classification of individuals as based on their BMD (osteoporotic, osteopenic or normal) and TBS (degraded, partially degraded or normal) values provides a better understanding of the bone’s resilience to fracture and enables a clinician to make a well-informed decision on patient management. However, 75% of women and 90% of men with a high risk of osteoporosis are not referred to a bone specialist for proper management [9]. To overcome the necessity of pro-activeness to detect individuals at high fracture risk, Medimaps group has recently developed a deep-learning approach, namely the “TBS Reveal” model. TBS Reveal is a multi-stage artificial neural network (ANN) architecture that can screen bone’s fragility from conventional/digital X-rays, and flag individuals at very high fragility who could be referred to bone specialists for full bone health assessment, including DXA.

Prior studies have shown promising results for the estimation of the BMD from conventional/digital X-ray and CT images using similar supervised learning approaches [11], but without taking into account the bone structure as assessed by TBS.

The overall aim of this study is to develop and externally validate the TBS Reveal model on conventional spine radiographs, and consider further improvements to expand its application to fracture risk assessment.

**Primary endpoint**

**To evaluate and validate TBS Reveal model’s performance on spine radiographs, including its vertebral segmentation and bone fragility prediction ANN architectures.**

The primary endpoint of this project collaboration consists of training, testing and externally validating the overall performance of the model in predicting bone fragility, based on the combined reference standards BMD T-score and TBS categories, using spine radiographs.

The reference standard will be derived from DXA, using the latest version of TBS iNsight v4.0 that incorporates a soft-tissue correction based on DXA-measured soft-tissue thickness.

The very high bone fragility category will be defined as the combined osteoporosis and degraded bone status from reference standard measures.

The aim is to develop and validate a deep-learning framework to predict whether input radiographs indicate very high bone fragility. Model development will use a combined multi-site dataset with an 80/10/10 random split for training, validation, and internal testing, stratified for the very high fragility class and with no subject overlap to prevent data leakage. Final model performance, generalizability, and robustness will be assessed using the internal test set and independent external cohorts collected a posteriori. External validation will, where possible, include diverse ethnic populations to evaluate robustness across populations.

**Secondary endpoint**

**To evaluate the associations between TBS Reveal predictions and fracture status, including both prevalent and incident fractures.**

We aim to clinically validate TBS Reveal with Fracture Outcomes, to assess the associations between the predicted bone fragility from conventional radiographs, with fracture outcomes. Fracture outcomes can be seen as prevalent fractures, to demonstrate fracture discrimination capabilities, or incident fractures to evaluate the predictive performance for future fractures.

**Images and data**

**All data are to be de-identified but connected with a patient ID.**

**Inclusion criteria for the study population:**

1. *Sex*: men and women
2. *Ethnicity*: all ethnicities (ethnicity identified in the data file where possible / applicable)
3. *Interval*: ≤ 6 months in between an individual DXA scan and a radiograph
4. Type of DXA and radiographs should be pairs of:

- **DXA scans - Lumbar spine, femur/hip, and/or 1/3rd radius and VFA images;**
- **Conventional/digital radiographs** - all views of lumbar vertebrae in AP/PA views, for example, AP lumbar, AP_thoracic, chest, pelvis, abdomen. Resolution ≤ 0.2 mm per pixel.

**- Each pair of images must be associated with the same individual and acquired within 6-months**
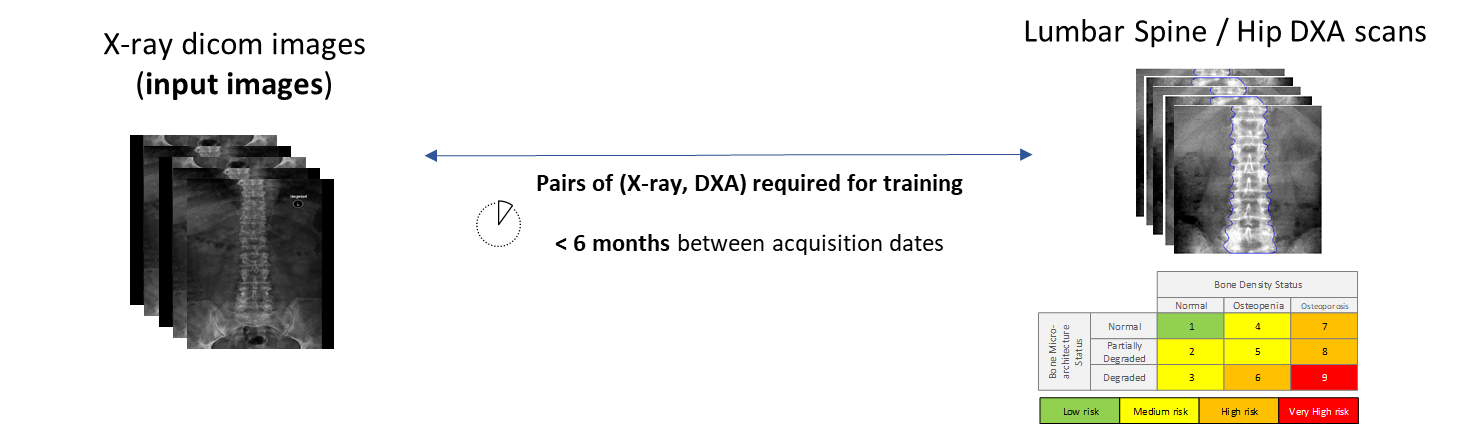


**Scan images - technical requirements:**

As both DXA scan files and DICOM conventional radiograph files include meta-data aside of the image, it is important to share de-identified scan files while preserving some of the required features.

- DXA scans: in manufacturer file format including non-sensitive meta-data (the following identifier**ⁱ**, Birthdate proxy**ⁱⁱ** and Acquisition date proxy**ⁱⁱ**)

- BMD, acquisition parameters, from compatible DXA devices:

- **GE*:*** GE Lunar Prodigy series (Primo, Pro, Advanced), GE Lunar iDXA series. Scan modes: standard, thick, thin and one scan.
- **Hologic:** Horizon A, C, W, Ci, Wi; Discovery A, C, W, SL; QDR 4500 A, C, W, SL; Delphi A, C, W, SL. Scan modes: array, fast array and high definition.
- Conventional radiographs: in the DICOM standard file format including non-sensitive meta-data (Anonymized identifier**ⁱ**, Birthdate proxy**ⁱⁱ**, acquisition parameters, acquisition date proxy**ⁱⁱ**)
- Conventional radiographs from any vendor, ≤ 0.2 mm per pixel resolution

***ⁱ*** *should be the same in both scan types*

***ⁱⁱ*** *Individual’s dates included as pseudo-anonymized (Year of birth date is kept, Day and Month will be generic)*

**Clinical data requirements**

The following clinical data should be provided in a separate file (e.g. Excel spreadsheet or text/csv file with separator indication), enabling the association of a scan file name with corresponding data. An identifier shall be generated by the participating clinical site to allow the association of DXA scan file(s), radiographs and clinical attributes per individual. Names must be removed. The desired clinical attributes are:

- Patient Identifier - generated for the study
- Proxy of Birthdate and acquisition date
- BMD and/or BMD T-Scores (LS, Total Hip, Femoral Neck, 1/3^rd^ Radius) of each DXA scan
- Sex
- Ethnicity
- ***If available – secondary endpoint:***
  - Vertebral fracture status (yes/no)
  - BMI
  - FRAX Clinical risk factors and diabetes status (yes/no)
  - Vertebral fracture level
  - Vertebral fracture grading
  - Femur/hip fracture status (yes/no)
  - Pelvis fracture status (yes/no)
  - Forearm fracture status (yes/no)
  - Humerus fracture status (yes/no)

**Data transfer process**

**Pseudo anonymization**

For data transfer specifications, it is important to remove personal identifiers. A pseudo anonymization tool (PSAT; developed by Medimaps Group, Geneva, Switzerland), handles de-identification of sensitive data and can be provided for DXA scans (manufacturer files) and conventional radiographs (.dcm files) to support participating clinical sites. Through a user interface, this tool allows selecting fields to de-identify fields such as Patient Identifier, Dates, First Name, Last Name, Patient ID, Height, Weight, etc. in a recursive and convenient way.

**Secured data transfer**

Data transfer will be ensured using a secured file transfer protocol, where every collaborator will have a dedicated account with login and password, for sharing data or software tools with each other. Detailed information for account creation will be provided after the study participation agreement has been signed and received.

**References**

[1] GBD 2019 Fracture Collaborators. Global, regional, and national burden of bone fractures in 204 countries and territories, 1990-2019: a systematic analysis from the Global Burden of Disease Study 2019. Lancet Healthy Longev. 2021 Sep;2(9):e580-e592. doi: 10.1016/S2666-7568(21)00172-0. PMID: 34723233; PMCID: PMC8547262.

[2] John A. Kanis JA,. et al.. SCOPE 2021: a new scorecard for osteoporosis in Europe. Archives of Osteoporosis (2021) 16:82.

[3] Kanis JA., et al. Long-term risk of osteoporotic fracture in Malmö. Osteoporosis international: a journal established as result of cooperation between the European Foundation for Osteoporosis and the National Osteoporosis Foundation of the USA. 2000;11(8):669-74.)

[4] Dempster DW. Osteoporosis and the burden of osteoporosis-related fractures. Am J Manag Care. 2011 May;17 Suppl 6:S164-9. PMID: 21761955.

[5] World Health Organization. (‎1994)‎. Assessment of fracture risk and its application to screening for postmenopausal osteoporosis : report of a WHO study group [‎meeting held in Rome from 22 to 25 June 1992]‎. World Health Organization. https://apps.who.int/iris/handle/10665/39142

[6] (Hernlund, E., et al., Osteoporosis in the European Union: medical management, epidemiology and economic burden. A report prepared in collaboration with the International Osteoporosis Foundation (IOF) and the European Federation of Pharmaceutical Industry Associations (EFPIA). Arch Osteoporos, 2013. 8: p. 136;

[7] Solomon DH., et al. Osteoporosis medication use after hip fracture in U.S. patients between 2002 and 2011. J Bone Miner Res. 2014;29(9): 1929–1937

[8] Kanis JA., et al. SCOPE 2021: a new scorecard for osteoporosis in Europe. Archives of Osteoporosis (2021) 16:82. Bone health and osteoporosis: a report of the Surgeon General. - Rockville, Md.: U.S. Dept. of Health and Human Services, Public Health Service, Office of the Surgeon General; Washington, D.C.: For sale by the Supt. of Docs., U.S. G.P.O., 2004. p.436)

[9] Nguyen TV, Center JR, Eisman JA. Osteoporosis: underrated, underdiagnosed and undertreated. Med J Aust. 2004 Mar 1;180(S5):S18-22. doi: 10.5694/j.1326-5377.2004.tb05908.x. PMID: 14984358.

[10] Hans D, Goertzen AL, Krieg MA, Leslie WD. Bone microarchitecture assessed by TBS predicts osteoporotic fractures independent of bone density: the Manitoba study. J Bone Miner Res. 2011 Nov;26(11):2762-9. doi: 10.1002/jbmr.499. PMID: 21887701.

[11] Zhang B., et al. (2020). Deep learning of lumbar spine X-ray for osteopenia and osteoporosis screening: A multicenter retrospective cohort study. Bone. 10.1016/j.bone.2020.115561.
